# The SNABB ADHD treatment scale—An easy-to-use scale on treatment monitoring in childhood ADHD: A pilot study

**DOI:** 10.3389/frcha.2023.1114565

**Published:** 2023-04-05

**Authors:** Elin Nylander, Jonas Hermansson, Gudrun Nygren

**Affiliations:** ^1^ Research Department, Angered Hospital, SV Hospital Group, Gothenburg, Sweden; ^2^ Child and Adolescent Specialist Centre, Angered Hospital, SV Hospital Group, Gothenburg, Sweden; ^3^ Gillberg Neuropsychiatry Centre, Sahlgrenska Academy, University of Gothenburg, Gothenburg, Sweden

**Keywords:** ADHD rating scales, child ADHD, attention deficit hyperactivity disorder (ADHD), treatment monitoring, response to treatment, teacher ratings, parent ratings

## Abstract

**Introduction:**

Information from parents and teachers are essential in the treatment monitoring of children with attention deficit hyperactivity disorder (ADHD). Rating scales are infrequently used in the treatment monitoring, and clinicians are signalling logistic barriers in the administration of rating scales in clinical settings. Here, we aimed to try out a new easy-to-use scale to facilitate information sharing between parents, teachers, and medical staff, in the treatment of childhood ADHD.

**Methods:**

We examined the SNABB scale in a clinical sample of 27 child- and adolescent patients with any type of ADHD, in a routine clinical setting. We compared the outcome of the new SNABB scale with the commonly used Swanson, Nolan, and Pelham Teacher and Parent ADHD rating scale—version IV (SNAP-IV).

**Results:**

The SNABB questions concerning ADHD cardinal symptoms hyperactivity and impulsivity were associated with the concurrent SNAP-IV subscale, with moderate to strong correlations. The SNABB inattention question failed all associations with the concurrent SNAP-IV inattention subscale. A secondary finding was that the SNABB mood regulation question correlated with the SNAP-IV ODD-subscale at all three measure points.

**Conclusion:**

Present pilot study brings promising results for the possibility to carry out larger scale studies concerning the psychometric properties of the SNABB scale.

## Introduction

The neurodevelopmental disorder (1) attention deficit hyperactivity disorder (ADHD) is one of the most common diagnoses in child and adolescent psychiatry today, with a prevalence in the range of 3%–7% (2–5). The clinical presentation of ADHD across ages is heterogenous and often includes symptoms outside the diagnostic ADHD frame, such as sleep problems (6), obesity (7), or emotional dysregulation (8). Co-morbid ADHD subgroups differ in severity of symptoms and functioning level, which are important factors in the planning and monitoring of treatment (9). Even though stimulants help improve symptoms of ADHD, some patients might still experience symptoms or functional deficits, causing a need for further treatment interventions (10, 11).

Information from parents and teachers is essential in the treatment monitoring of childhood ADHD, even though different informants (e.g., parents, teachers) differ in their expectations and tolerance against different behaviors and therefore might disagree on the nature of the problems (12, 13). Many psychiatric conditions, including ADHD, are context-dependent and demonstrate different symptoms depending on the specific situation (12). For example, the most common contexts for children are the home and school environments, where the classroom situation might be more challenging and hence more revealing for children exhibiting symptoms of ADHD than the home environment. This calls for a multi-informant perspective, to get a comprehensive picture of each patient. However, experts seem to differ as to how reports from parents and teachers show these differences between environments or not (14–17).

Numerous rating scales on the symptoms of childhood ADHD have been developed over the years (18). Rating scales are clinically useful in the assessment phase as well as in assisting treatment planning and monitoring and supporting treatment responses (19). Most studies on ADHD treatment outcomes focus on the reduction of core ADHD symptoms alone, despite most patients with ADHD exhibiting symptoms outside the diagnostic criteria (8) and many children and adolescents with ADHD experiencing problems with their overall health and wellbeing (20). Epstein and Weiss (21) suggest that aspects of executive functions, overall functional impairment, quality of life, and adaptive life domains are important factors to include in the monitoring of ADHD treatment responses.

Unfortunately, these rating scales in the monitoring of treatment response are used infrequently (22). For example, rating scales were only completed by 7.5% (teachers) and 10.8% (parents) for the patients during their first year of ADHD treatment in one study (23) and were not used at all in another (24). With respect to this, there are multiple logistic barriers in the use of rating scales for clinicians to overcome in order to improve treatment monitoring for children with ADHD (19). First, clinicians seldom have direct contact with teachers and school staff to receive first-hand information about the child's functioning and symptoms (19). Second, clinicians have limited time for each appointment, often leading to parents filling out rating scales at home without guidance, explanations, or reminders of bringing them back to the clinic for evaluation. Third, rating scales sometimes use a specific, medical language that is difficult for laymen to understand and thereby jeopardizes the reliability in the answers.

The primary aim of the present study was to improve the information sharing between medical staff, parents, and school staff, in the monitoring of ADHD treatment in child and adolescent patients, by trying out a pilot version of an easy-to-use scale developed at a child and adolescent specialist center by clinicians. The SNABB (“snabb” simply refers to the Swedish word for “quick”) scale includes ratings of parental concern about common problems in the treatment of ADHD beyond the sole focus on core ADHD symptomatology. The secondary aim of the study was to describe the outcomes of the SNABB scale in terms of clinical usability when monitoring treatment response in a clinical sample, but also to compare the outcome of the three questions in the SNABB scale concerning core symptoms of ADHD to the corresponding questions in the commonly used Swanson, Nolan, and Pelham Teacher and Parent ADHD rating scale—version IV (SNAP-IV) ADHD symptom rating scale (9, 25, 26).

## Materials and methods

### Patient selection

At a Swedish child and adolescent specialist center, at the Angered Hospital, in a suburb of Gothenburg, Sweden, we included all patients with ADHD starting treatment between September 2019 and May 2020 who consented to participation. This led to the participation of 27 consecutive patients with ADHD , of whom 20 were boys. The age range of the patients was 6–17 years (median 11 years). For further details on the study sample, see Table 1. All patients were diagnosed with any type of ADHD (the ADHD subtypes were not registered for the purpose of the present study as the treatment outcome was not the focus here).

**Table 1:** Descriptive statistics of the 27 patients presented in *N* (percentages) and where applicable, in medians and interquartile range (IQR).

	*N* (%)	Median	IQR
Boys	20 (74)	–	–
Girls	7 (26)	–	–
Boys age	–	11	3.75
Girls age	–	12	4
Total age	–	11	3
FSIQ^a^	–	88^b^	23
FSIQ ≥ 90^c^	13 (48)	–	–
FSIQ < 90^d^	14 (52)	–	–
Comorbid developmental disorders^e,f^	17 (63)	–	–
Comorbid affective disorders^e,g^	5 (19)	–	–
Sleep problems *before* treatment^e^	12 (44)	–	–
Sleep problems *during* treatment^e^	11 (41)	–	–
Eating problems *before* treatment^e^	4 (15)	–	–
Eating problems *during* treatment^e^	5 (19)	–	–
Treatment Centralstimulantia^e^	23 (85)	–	–
Treatment other ADHD-drug^e^	2 (7)	–	–
Informant mother^h^	15 (56)	–	–
Informant father^h^	2 (7)	–	–
Informant both parents^i,h^	10 (37)	–	–

^a^ Full Scale Intelligence Quotient.

^b^ For two patients only range scores were reported, for those the mean of specified range was calculated and included.

^c^ Average or above.

^d^ Low average or below average.

^e^ According to medical records.

^f^ Autism spectrum disorder (ASD), developmental coordination disorder (DCD), dyslexia.

^g^ Depression, anxiety disorders.

^h^ At baseline.

^i^ Informant not further specified.

The patients included in the present study were following routine clinical practice at the child and adolescent specialist center for their ADHD treatment; we simply added the SNABB scale to the routine. In this routine clinical practice, the patients had an appointment with a physician during the ADHD assessment, and thereafter continual follow-ups with a nurse specializing in neuropsychiatric treatment, if drug treatment was initiated. In the present sample of 27 patients with ADHD, 23 (85%) were medicated with stimulants (CS), two (7.5%) were medicated with other ADHD drugs, and the remaining two (7.5%) were unmedicated during the study period.

Before the start of the study, we aimed to use three measuring points for the SNABB scale: at the start of treatment and 1 and 3 months later. However, due to unforeseen factors, such as the COVID-19 pandemic, and everyday clinical practice being affected by multiple possible confounders present at any given time, the time elapsed between the follow-ups varied among the 27 patients. However, as the treatment outcome was not the focus of the present study, this was deemed to be of negligible significance.

Considering the parent reports, 14 of the 27 patients participated in the first of the two follow-ups, and 11 of the 27 patients participated in the second follow-up. Thus, only 8 patients participated in both the baseline and the two follow-ups, while 10 patients only participated in the baseline rating and were considered lost to follow-up; for these patients, an attrition analysis was conducted (vide infra). In the parent ratings, the informants were mostly mothers alone (*n*=15, 56%) or both parents together (*n*=10, 37%). No information about parental psychopathology was collected for the purpose of the present pilot study.

As for the teacher ratings, 6 of the 27 patients were missing teacher ratings; only 8 patients had all three teacher ratings completed. The remaining 13 patients had one or two teacher ratings completed. No attrition analysis was conducted for missing teacher reports.

For complementary information concerning the treatment process, the medical records were scrutinized by an experienced physician specializing in child psychiatry (GN) who was well familiar with the instruments used in the present study, as well as with the clinical procedure in the ADHD assessment at the clinic where the data were collected.

Finally, we are aware of there being a sizable group of patients with ADHD treated at the clinic during the study period who were not included in the present study. This has to do with multiple factors, such as the parents or patient declining participation in the study, missing information about participation status, or patients diagnosed before the start of the study. Due to ethical principles, it is not possible to further examine this group outside the study sample. However, 10 patients (median age 12 years) were lost to follow-up in the present study; they only participated in the parent baseline rating. According to their medical records, six patients were sent questionnaires to complete at home, yet did not return them to the clinic; the remaining four patients were either remitted to another clinic or dropped out of treatment (during the chosen time span).

### Instrument design and selection

The SNABB scale (see Supplementary Material for both the Swedish and English versions, the last for illustrative purposes) was developed by clinicians at a child and adolescent specialist center, during a collaborative project (Swedish: “Skolsamverkan”) between the same child and adolescent specialist center and an elementary school in the suburbs of Gothenburg, Sweden. The clinicians in this study saw the need for a new easy-to-use instrument, independent of linguistic and cultural background, that could facilitate information sharing in the assessment of the response to medical treatment in child ADHD. The questions in the SNABB scale focus on the parental concern about the cardinal symptoms of ADHD and common comorbidity and/or side effects to medical treatment. The instrument contains six questions, here named A–F. All questions are answered by highlighting the number on a Likert scale of 0–10 that best corresponds to the degree of difficulty, where 0 indicates no concern and 10 great concern. The SNABB questions A–C measure the three cardinal symptoms in ADHD (A hyperactivity, B impulsivity, and C inattention). The fourth question, D, was added to rate mood regulation problems that are a common add-on to the ADHD symptomatology (8). Parents rate the degree of parental concern for the child's mood (e.g., symptoms of depression, anger, or temper tantrums). The SNABB question E, concerning eating problems, was added as reduced appetite is a common side effect of CS treatment and because child ADHD often comes with problems regulating food intake (and with an elevated risk of developing obesity (7). There was also a need for a question concerning sleeping problems, question F, as these problems are common in child ADHD (6), and sometimes worsen with CS treatment. In the clinic, and thereby also in the study, the definition of sleeping problems was spending >1 h awake in bed before falling asleep and/or one or multiple periods awake during the night. For about one-third of the present sample, medical treatment with melatonin was initiated before or along with the CS treatment. Further, the SNABB scale offers two open-ended questions linked to sleeping and eating problems. A total of 20 (74%) parents used the opportunity to comment on the baseline rating in the present study; this might signal a need to comment on the numeric ratings of symptoms. In total, 22 (82%) parents estimated the time consumed to answer the SNABB instrument to be less than 5 min. Before the start of the ADHD treatment, the parents were given explanations to questions A–F (Supplementary Material), if necessary, *via* an interpreter. An information letter was sent to each teacher who participated. The focus on ratings from school was on questions A–C. The estimation of sleep could be omitted if no specific information was available or could be commented on in free text, e.g., fatigue during the school day. As the children had lunch at school, there was also an opportunity to estimate the food situation at school.

To place the SNABB scale in the context of methods of quantifying the symptoms of ADHD, an additional scale was used. The validated SNAP-IV is a commonly used scale in ADHD symptom rating, screening, and treatment research (26). The SNAP-IV was already an implemented screening tool regularly used in the ADHD assessments and treatment monitoring at the clinic where this study was performed. The original SNAP consists of 43 items but was shortened to 26 items for use in the Multimodal Treatment of Attention Deficit Hyperactivity Disorder study (MTA study) (27). The shorter version consists of the DSM-IV ADHD and oppositional defiant disorder (ODD) symptoms. SNAP-IV is constructed as a 4-point Likert scale where the parent (or teacher) rates the child's symptoms as 0 (not at all), 1 (just a little), 2 (quite a bit), or 3 (very much), with total scores in the range of 0–90. The items can be separated into three subscales: Inattention (items 1–9); Hyperactivity/Impulsivity (items 11–19); and ODD (items 21–28). There is a clinical 5% cutoff in the SNAP-IV, separated by parents (Inattention: 1.78, Hyperactivity/Impulsivity: 1.44, and ODD: 1.67) and teachers (Inattention: 2.56, Hyperactivity/Impulsivity: 1.78, and ODD: 1.67). The cutoff refers to the top 5% extreme scorers (≥95th percentile).

The test–retest reliability for SNAP-IV was .66–.92, according to Swanson et al. (28). The internal consistency for the SNAP-IV parent version was .94 while the teacher version was .97 (25). Overall, the SNAP-IV reliability was acceptable according to Bussing et al. (25), with a moderate inter-rater reliability. Ullebø et al. (17) found, in their factor analysis of SNAP-IV, support for the theory of a strong general ADHD factor and concluded that their results support the view that ADHD was a unitary concept. SNAP-IV showed a high sensitivity but low specificity to the clinical diagnosis of ADHD (26) and is considered by the same author to be a valid measure in the clinical setting even though it is best viewed as a screening tool rather than a comprehensive diagnostic scale for ADHD. SNAP-IV has also been criticized for lacking published data on psychometric properties (18). However, SNAP-IV has been used in Swedish samples before (9).

### Statistical analyses

The descriptive data in the tables are presented as numbers, percentages, medians, and interquartile ranges (IQR), unless noted otherwise. We chose non-parametric tests because of their robustness to the violence of normality and because others (29) have encouraged the use of non-parametric tests in psychiatric studies like ours. Associations between the variables in the two instruments were computed as Spearman correlations, and due to the small sample size in the present work, an association of ≥.6 was considered relevant. Within-question/subscale differences were assessed using the Wilcoxon signed-rank test. Here, we compared the baseline scores with scores at the second follow-up, separated by parents and teachers, and for each SNABB question and each SNAP-IV subscale separately. Effect sizes are expressed as partial eta-squared (*η*^2^), and the computation of *η*^2^ following the Wilcoxon signed-rank tests (for both SNABB questions and SNAP-IV subscales) employed an online calculator (https://www.psychometrica.de/effect_size.html). The magnitude of *η*^2^ is expressed as small (.01), medium (.06), or large (.14) (30). For the 10 patients who were lost to follow-up, an attrition analysis was conducted using descriptive statistics and the Mann–Whitney *U* test. SPSS Statistics for PC version 29.0 (IBM, Armonk, NY, USA) was used for all calculations.

## Results

### Parent and teacher SNABB and SNAP-IV ratings at baseline and at follow-ups

Table 2 shows the medians and IQRs of the parent- and teacher-rated SNABB and SNAP-IV, at the three measuring points separately. According to the SNAP-IV parent scores, the patients had, at a group level, symptoms above cutoff at baseline for the hyperactivity/impulsivity and the inattention scales (Table 2). At the final, second, follow-up, the parents rated their children significantly improved for the SNAP-IV inattention scale (*Z* = −2.43, *p* = .015, *η*^2^ = .59) but not for the SNAP-IV hyperactivity/impulsivity scale (*Z* = −1.86, *p* = .063). However, the children were improved according to the parent ratings on the SNABB scale for the questions concerning impulsivity (*Z* = −2.11, *p* = .035, *η*^2^ = .41) and inattention (*Z* = −2.21, *p* = .027, *η*^2^ = .44), yet not concerning hyperactivity (*Z* = −1.21, *p* = .227). For the teacher ratings none of the SNAP-IV ratings were elevated according to the clinical cutoff, and none of the SNAP-IV subscales were significant by the Wilcoxon signed-rank test (*Z* = −1.52 and −1.90, *p* > .05).

**Table 2:** Descriptive statistics (medians and interquartile ranges; IQR) for all SNABB questions separately and for the two SNAP-IV clusters inattention and hyperactivity/impulsivity, separated by parent or teacher ratings, at the three measure points (at baseline and at the two follow-ups).

	SNABB A hyperactivity		SNABB B impulsivity		SNABB C inattention		SNABB D mood regulation problems		SNABB E eating problems		SNABB F sleeping problems		SNAP-IV inattention		SNAP-IV hyperactivity/impulsivity		SNAP-IV ODD	
	Median	IQR	Median	IQR	Median	IQR	Median	IQR	Median	IQR	Median	IQR	Median	IQR	Median	IQR	Median	IQR
Baseline																		
Parents (*N* = 26–27)	7	4	7	4	8	3	6	3	5	6	6	6	**2**.**11**^a^	0.81	**1**.**73**^a^	0.84	**1**.**69**^a^	0.91
Teachers (*N* = 13–21)	6	4.75	3	7	8	2	4	7	1	5	1	7.5	2.11	1.11	0.89	1.95	0.63	1.19
Follow-up 1																		
Parents (*N* = 14)	2.5	3.75	3	3.75	5.5	3	4	3.25	2.5	5	1.5	3.25	1.45	1.47	0.73	0.73	0.82	1
Teachers (*N* = 6–12)	3	4	2	2.75	6.5	4	1.5	5	1	2.75	1	2.75	1.45	1.22	0.22	0.86	0.19	0.85
Follow-up 2																		
Parents (*N* = 11)	4	5	3	4	6	4	5	7	5	8	3	4	1.33	1	0.89	0.67	1	1.75
Teachers (*N* = 4–9)	5.5	5.5	2.5	5.5	7	5	2	8.25	2.5	9	0.5	4	1.78	1.34	0.22	1.83	0.25	1.38

SNAP-IV values above cutoff are highlighted as bold.

^a^ Value >5% clinical cut-off (>95th percentile).

In line with this, the teacher ratings of hyperactivity (SNABB A) and impulsivity (SNABB B) also came out as non-significant by the Wilcoxon signed-rank test (*Z* = −.09 and −.41, *p* > 0.5), yet for the SNABB C (inattention) there was a significant improvement from baseline to the second follow-up (*Z* = −1.98, *p* = .048, *η*^2^ = .49). However, the medians of the teacher SNAP-IV reports were below the clinical cutoff, indicating milder or manageable symptoms in the school setting, according to teachers.

In addition, the questions concerning mood regulation (SNABB D), eating problems (SNABB E), and sleeping problems (SNABB F), rated by parents and teachers, failed the Wilcoxon signed-rank test (*Z* = −0.22 to −1.10, *p* > .05), except for the parent-rated sleeping problems (SNABB F) where the change from baseline to the second follow-up came out as significant (*Z* = −2.15, *p* = .03, *η*^2^ = .42). This indicates improved sleep for the child/adolescent since the start of treatment, according to parents.

### Associations between SNABB and SNAP-IV score

Tables 3 and 4 show the Spearman correlation coefficients between SNABB scores on the one hand and SNAP-IV scores on the other, separated by parent (Table 3) and teacher (Table 4) ratings.

**Table 3:** Parent ratings of core ADHD symptoms in SNABB and SNAP-IV, at baseline and two follow-ups, correlated using Spearman's *r* (*N* = 8–27).

	SNAP-IV inattention	SNAP-IV hyperactivity/impulsivity	SNAP-IV inattention	SNAP-IV hyperactivity/impulsivity	SNAP-IV inattention	SNAP-IV hyperactivity/impulsivity
	Baseline	Baseline	Follow-up 1	Follow-up 1	Follow-up 2	Follow-up 2
Baseline						
SNABB A	.363	.522**				
SNABB B	.518**	.693**				
SNABB C	.512**	−.066				
Follow-up 1						
SNABB A			.390	.770**		
SNABB B			.295	.714**		
SNABB C			.535*	.177		
Follow-up 2						
SNABB A					.599	.817**
SNABB B					.508	.865**
SNABB C				-	.327	.189

SNABB A concerns hyperactivity, SNABB B concerns impulsivity and SNABB C concerns inattention. Significant correlations ≥.6 are highlighted as bold.

* Correlation is significant at the 0.05 level (two-tailed).

** Correlation is significant at the 0.01 level (two-tailed).

**Table 4:** Teacher ratings of core ADHD symptoms in SNABB and SNAP-IV at baseline and two follow-ups, correlated using Spearman's R (*N* = 7–20).

	SNAP-IV inattention	SNAP-IV hyperactivity/impulsivity	SNAP-IV inattention	SNAP-IV hyperactivity/impulsivity	SNAP-IV inattention	SNAP-IV hyperactivity/impulsivity
	Baseline	Baseline	Follow-up 1	Follow-up 1	Follow-up 2	Follow-up 2
Baseline						
SNABB A	.448*	.720**				
SNABB B	.376	.833**				
SNABB C	.502*	.366				
Follow-up 1						
SNABB A			.423	.202		
SNABB B			.289	.638*		
SNABB C			.598*	−.032		
Follow-up 2						
SNABB A					.729*	.209
SNABB B					.548	.957**
SNABB C					.247	.013

SNABB A concerns hyperactivity, SNABB B concerns impulsivity and SNABB C concerns inattention. Significant correlations ≥.6 are highlighted as bold.

* Correlation is significant at the 0.05 level (two-tailed).

** Correlation is significant at the 0.01 level (two-tailed).

At baseline, only the SNABB B (impulsivity) item correlated positively with the concurrent SNAP-IV rating for parents (Table 3); for teachers, the SNABB A (hyperactivity) and SNABB B (impulsivity) correlated positively with the concurrent SNAP-IV ratings (Table 4). The correlations were in the moderate to strong range (*r* = .693–.833). The SNABB C (inattention) parent and teacher ratings were not associated with the concurrent SNAP-IV subscale at any of the three measuring points when applying the stricter model of relevant correlations (≥.6) (Tables 3, 4). Overall, for the two follow-ups, the correlations were somewhat fewer, but still in the moderate to strong range. The SNABB B (impulsivity) and the SNAP-IV hyperactivity/impulsivity correlated positively through all three measuring points for both parents and teachers, with moderate to strong correlations. The SNABB A (hyperactivity) was significantly associated with its SNAP-IV counterpart (inattention) only at the second teacher follow-up (not at baseline or the first follow-up) (Table 4).

A question arising during this work concerned the relation between the SNABB D (mood regulation) and the SNAP-IV ODD subscale; in an exploratory manner, we conducted Spearman's *r* for each time point, separated by parents and teachers, with the following results: parent ratings: baseline (*n* = 26), *r *= .658, *p* < .001; follow-up 1 (*n* = 14), *r* = .657, *p* = .011; and follow-up 2 (*n* = 11), *r* = .954, *p* < .001; and teacher ratings: baseline (*n* = 21), *r* = .743, *p* < .001; follow-up 1 (*n* = 12) *r* = .758, *p* = .004; and follow-up 2 (*n* = 8) *r* = .767, *p* = .026. Thus, the SNABB D mood regulation and the SNAP-IV ODD subscale were significantly associated at each of the three time points, for both parents and teachers.

## Discussion

The aim of the present pilot study was to try out a new scale for the monitoring of child ADHD treatment. The scale included questions distilled out of clinical experience with the purpose of mirroring clinical reality. The study was conducted at a child and adolescent specialist center in a socioeconomically disadvantaged area with a multiethnic population, which was also an inspiration when creating the SNABB scale, as we have often experienced that language barriers with parents is an issue. More specifically, we wished to improve the information sharing between medical staff, parents, and school staff, in the monitoring of ADHD treatment in child and adolescent patients. The SNABB scale examines parental concerns about the common problems in the treatment of ADHD beyond the sole focus on core ADHD symptomatology.

The main finding of this study was that the SNABB questions regarding the ADHD symptoms hyperactivity and impulsivity were associated with the concurrent subscale from the well-established SNAP-IV ADHD rating scale (Tables 3, 4), in both parent and teacher ratings, with moderate to strong correlations as significant *r*s were in the range of .638–.957 ( *p* < .05). Contrarily, the SNABB question concerning inattention was not associated with the concurrent subscale of inattention in the SNAP-IV. This was perhaps because externalized symptoms are easier for parents and teachers to notice than internalized inattentive problems, as externalizing symptoms are more intrusive and tend to cause more problems around the child (with peers, in the classroom, in the family, etc.) (31, 32). In addition, the SNABB inattention question does not provide information about different aspects of inattention symptoms, nor was it designed to do so. However, the results from the present study could also be due to the lack of statistical power in the statistical analyses considering the few included participants. With regard to that, these findings should be carefully interpreted considering the small sample size, yet these interesting first findings clearly pave the way for further examinations in larger samples of the SNABB scale.

With respect to the SNABB questions regarding mood regulation, sleep, and eating problems, only the question about sleep problems survived the Wilcoxon signed-rank test with a large effect size (*η*^2^ = .42), indicating improved sleep according to parents and teachers. With regard to this, just under half (12 patients) of the pilot sample of 27 patients had reported sleep problems before the start of the study (according to the medical records) and at least 10 of these remained bad sleepers at the final follow-up (according to the medical records), several months after the initiation of medical treatment, yet were improved at a group level (according to the SNABB reports), possibly due to the ongoing medication with melatonin. However, since almost 50% of the present study sample reported sleep problems, it is a central matter in the monitoring of ADHD treatment. Thus, sleep problems in child ADHD are not news, as they have been reported in as many as 70% of cases (6), and often continue into adulthood (8). Further, the addition of open-ended questions connected to the SNABB questions about sleep (and eating problems) was successful, as 74% of the parents chose to extend the numeral rating with a further explanation within their 5 min spent completing the questionnaire. These comments bring further dimensions to the information sharing and are of great importance in the clinical work. However, with the reported symptoms, one must keep in mind that the reporting most likely mirrors an accumulation of long-term symptoms (academic, social skills, etc.) rather than the isolated current situation (33), all leading up to the clinical presentation.

An interesting secondary finding in the present study is the fact that the SNABB D scale concerning mood regulation problems was associated with the SNAP-IV ODD subscale through all three measuring points (baseline and first and second follow-ups), in both parent and teacher ratings, according to Spearman's *r* correlations (*r*s in the range of .657–.954, *p* < .05). However, the significant association between the SNABB D and SNAP-IV-ODD subscale should be interpreted with caution and needs to be affirmed in larger sample studies. The SNABB D question catches the emotional dimension and mood regulation problems, but the oppositional dimension is not specifically included in the description of SNABB D. Clinical experience and the literature suggest that the emotional dimension and the oppositional dimension are not directly consistent. In the future validation of the SNABB, it might be important to consider the addition of a specific scale for oppositional defiant aspects. However, an easy-to-use scale, visual and without language barriers for the oppositional dimension might be a challenge.

This work was distilled out of professional experiences of difficulties in information sharing between medical staff and parents/teachers in the treatment of child ADHD, especially considering the administration of rating scales. Previous studies have noticed the presence of multiple logistic barriers in the administration of rating scales for clinicians (19), that rating scales are often infrequently used in treatment monitoring (22), and the lack of consensus regarding the most optimal format of reports (34). Barbaresi (19) suggests that a more effective use of rating scales would improve the treatment response in children with ADHD; if so, this is indeed an urgent matter in need of more attention. Concerning the rating scales for patients in healthcare in general, Khadka et al. (34) concluded that developers of patient-reported outcome scales should strive to use a simple question format with fewer response options (34). This is in line with the base upon which the SNABB scale was developed at its very beginning. The SNABB scale strives to facilitate information sharing, making it possible for clinicians to implement the use of the SNABB scale within the appointment. Hence, we, temporarily and for the purpose of the present pilot study, added a question concerning the time consumed on the SNABB scale. Of the participating parents, 82% reported the time consumed on the SNABB scale to be 5 min or less. The fact that the SNABB scale takes less than 5 min to complete makes it more likely to be included in appointments, making questions and explanations possible. This is important as reliable reporting only can be achieved when reporters understand the questions asked. If a rating scale used to collect data is improper in its administration, any conclusion based on those data will indeed be insecure (34).

A proposal for the future of the logistics concerning self-reports and rating scales, is to digitalize the procedure, as suggested by Barbaresi (19), making the procedure more effective for both reporters and clinicians. It would facilitate not only the clinical process but also enable first-hand information sharing with parents and teachers, and, for example, facilitate the possibility for adolescents to more easily self-report; for example, adolescents might be better reporters on their internal symptoms than their parents or teachers (22).

The strengths of this study include the careful diagnostic procedure that patients were passed through by an experienced collaborative team consisting of a physician, psychologist, special education teacher, and sometimes a speech therapist, at a child and adolescent specialist center. Another strength is the naturalistic study design, as patients followed the routine psychiatric care, adding the SNABB scale in the reporting procedure. Hence, the clinical origin, and the way SNABB reflects clinical everyday practice is perhaps the most important strength of the present study. Another advantage of the SNABB scale is its simplicity and visuality, making it likely to be more usable across cultural and linguistic barriers.

We are also aware of the limitations of this pilot study. First, the naturalistic study design is also a limitation as we had no control over treatment patterns, nor had we any control over the administration of the rating scales. For example, several patients were sent home with rating scales to bring back to the clinics, which increased the number of patients lost to follow-up or patients never returning their consent to participate, precluding them from participation. Another limitation is the small sample size, which, along with a new rating scale, hampers any firm conclusions. To handle this in the best way, we conducted non-parametric statistics, as it is more robust to violence and designed to handle small sample sizes. Hence, this pilot study is a first preliminary try-out of our new SNABB scale, and we hope the present study can serve as a starting point for future larger-scale studies of the usefulness and validation of the SNABB scale. We suggest that further studies on the SNABB scale be conducted with a separate focus on testing if the scale can aid in overcoming language barriers in clinical practice.

In conclusion, the SNABB scale questions concerning the ADHD cardinal symptoms of hyperactivity and impulsivity were associated with the concurrent subscale in the SNAP-IV, with moderate to strong correlations. The SNABB inattentive question failed all associations with the concurrent SNAP-IV subscale. The question of validity and reliability of the SNABB scale remains to be answered, yet the present pilot study brings promising results for the possibility of carrying out larger-scale studies concerning the psychometric properties of the SNABB scale.

## Data Availability

The raw data supporting the conclusions of this article will be made available by the authors, without undue reservation.
