# Supplementary material for: The SNABB ADHD treatment scale—An easy-to-use scale on treatment monitoring in childhood ADHD: A pilot study
Source: Front Child Adolesc Psychiatry. 2023 Apr 5;2:1114565. doi: 10.3389/frcha.2023.1114565 (PMC11732116; doi:10.3389/frcha.2023.1114565)
Supplement: Supplementary file 1 [file Datasheet1.pdf]

Föräldra- och lärarformulär för skattning av beteende, mat och sömn - SNABB

Datum:………………………………………………………………

Avser perioden:………………………………till:……………………………………..

Namn:………………………………………………………………..Pojke……Flicka…..

Personnummer:…………………………………………………………………………

Ifylld av:………………………………………………………………………………………..

Ringa in den siffra som bäst beskriver barnet

Aktivitetsnivå:

Ingen oro 0-----1-----2-----3-----4-----5-----6-----7-----8-----9-----10 Stor oro

Impulsivitet:

Ingen oro 0-----1-----2-----3-----4-----5-----6-----7-----8-----9-----10 Stor oro

Koncentration:

Ingen oro 0-----1-----2-----3-----4-----5-----6-----7-----8-----9-----10 Stor oro

Humör:

Ingen oro 0-----1-----2-----3-----4-----5-----6-----7-----8-----9-----10 Stor oro

Mat:

Ingen oro 0-----1-----2-----3-----4-----5-----6-----7-----8-----9-----10 Stor oro

Om oro finns, på vilket sätt?

____

Sömn:

Ingen oro 0-----1-----2-----3-----4-----5-----6-----7-----8-----9-----10 Stor oro

Om oro finns, på vilket sätt?

____

Är det något annat du vill tillägga?

____

____

Hur lång tid tog det att fylla i detta formulär? Ringa in det som passar bäst.

0-2 minuter 2-5 minuter 5-10 minuter

Förklaringar till formuläret_

Aktivitetsnivå:

Har barnet svårt att vara stilla med händer och fötter

Lämnar barnet sin plats i klassrummet eller i situationer där barnet förväntas sitta kvar

Springer barnet omkring, är barnet i ständig rörelse

Är barnet på språng

Impulsivitet:

Har barnet svårt att vänta på sin tur

Svarar barnet rakt ut

Avbryter eller stör andra

Gör barnet saker utan at tänka på vad som kan hända

Får barnet utbrott eller humörsvängningar

Koncentration:

Har barnet svårt med fokus och uthållighet i skolarbetet

Har barnet svårt att bibehålla uppmärksamheten i det han eller hon gör

Har barnet svårt att komma igång och göra färdigt uppgifter

Har barnet svårt att organisera sitt skolarbete

Humör:

Är barnet lättirriterat

Tappar barnet ofta humöret

Är barnet ofta argt eller lättstött

Har barnet svårt med krav

Är barnet ofta ledsen

Mat:

Äter barnet all mat eller endast vissa maträtter

Äter för mycket eller för litet

Sömn:

Har barnet svårt att somna

Går barnet och lägger sig i tid

Sover barnet hela natten

Trötthet under skoldagen
