# Supplementary material for: The SNABB ADHD treatment scale—An easy-to-use scale on treatment monitoring in childhood ADHD: A pilot study
Source: Front Child Adolesc Psychiatry. 2023 Apr 5;2:1114565. doi: 10.3389/frcha.2023.1114565 (PMC11732116; doi:10.3389/frcha.2023.1114565)
Supplement: Supplementary file 2 [file Datasheet2.pdf]

SNABB scale

Parent/ teacher rating scale on behaviour, feeding and sleep

Date of completion:……………………………………………………………………………………………………….

Concerns the following period:…………………………………….**to:**……………………………………….

Name of child:……………………………………………………………….**Gender**………………………………….

Personal identification number/date of birth:………………………………………………………….……

Completed by:……….

Circle the number that best describes the child:

A Activity level:

No concern 0-----1-----2-----3-----4-----5-----6-----7-----8-----9-----10 Great concern

B Impulsiveness:

No concern 0-----1-----2-----3-----4-----5-----6-----7-----8-----9-----10 Great concern

C Inattention:

No concern 0-----1-----2-----3-----4-----5-----6-----7-----8-----9-----10 Great concern

D Mood:

No concern 0-----1-----2-----3-----4-----5-----6-----7-----8-----9-----10 Great concern

E Feeding/Eating:

No concern 0-----1-----2-----3-----4-----5-----6-----7-----8-----9-----10 Great concern

Describe potential concern?

____

F Sleep:

No concern 0-----1-----2-----3-----4-----5-----6-----7-----8-----9-----10 Great concern

Describe potential concern?

____

Any additional comments?

____

____

How long did it take you to complete this questionnaire? Circle the best option.

0-2 minutes 2-5 minutes 5-10 minutes

Translated for illustrative purpose only

Explanations to questions A-E

(The explanations are given to the parents verbally before the start of ADHD treatment, if necessary, via an interpreter)

Activity level:

Does the child have issues keeping hands and/or feet still when needed?

Does the child leave his seat in the classroom or in situations where the child is expected to remain seated?

Is the child in constant movement, have difficulties being still?
Is the child always on the go?

Impulsiveness:

Dose the child have issues with waiting, standing in line?

Does the child answer directly without fully listening

Does the child interrupt och disturb other people?

Does the child act without taking in potential consequences?

Does the child have outbursts?

Inattention:

Does the child have issues with focus and perseverance in school?

Does the child have issues in maintain focus during different activities?

Does the child have issues in starting and completing tasks?

Does the child have issues regarding organising of school work?

Mood:

Is the child easily annoyed?

Does the child often lose its temper?

Is the child often angry or easily offended?

Does the child have issues with demands set by others on the child?

Is the child often sad or depressed?

Feeding/Eating:

Does the child eat a variety of foods or is the child very selective in food choice?

Does the child eat too much or to little?

Sleep:

Does the child have issues falling asleep?

Does the child go to bed/fall asleep on time?

Does the child sleep through the night?

Is the child tired or sleepy during school?

Translated for illustrative purpose only
